# Severe Acute Pancreatitis Treated with Negative Pressure Wound Therapy System: Influence of Laboratory Markers

**DOI:** 10.3390/jcm12113721

**Published:** 2023-05-28

**Authors:** Bogdan Mihnea Ciuntu, Dan Vintilă, Adelina Tanevski, Ștefan Chiriac, Gabriela Stefănescu, Irina Mihaela Abdulan, Gheorghe G. Balan, Bogdan Veliceasa, Oana Viola Bădulescu, Gabriela Ghiga, Ana Maria Fătu, Andrei Georgescu, Mihai Bogdan Vascu, Alin Mihai Vasilescu

**Affiliations:** 1 Department of General Surgery, “Grigore T. Popa” University of Medicine and Pharmacy, 700115 Iași, Romania; bogdan-mihnea.ciuntu@umfiasi.ro (B.M.C.); dan.vintila@umfiasi.ro (D.V.); papancea.adelina@umfiasi.ro (A.T.); alin.vasilescu@umfiasi.ro (A.M.V.); 2 Department of Gastroenterology, “Grigore T. Popa” University of Medicine and Pharmacy, 700115 Iași, Romania; stefanchiriac@yahoo.com (Ș.C.); gheorghe-g-balan@umfiasi.ro (G.G.B.); 3 Department of Medical Specialties I, “Grigore T. Popa” University of Medicine and Pharmacy, 700115 Iași, Romania; 4 Department of Traumatology and Orthopaedics, Faculty of Medicine, “Grigore T. Popa” University of Medicine and Pharmacy, 700115 Iași, Romania; bogdan.veliceasa@umfiasi.ro; 5 Department of Haematholohy, Faculty of Medicine, “Grigore T. Popa” University of Medicine and Pharmacy, 700115 Iași, Romania; viola.badulescu@umfiasi.ro; 6 Department of Mother and Child Medicine, Faculty of Medicine, “Grigore T. Popa” University of Medicine and Pharmacy, 700115 Iași, Romania; gabriela.ghiga@umfiasi.ro; 7 Discipline of Ergonomy, Department of Implantology Removable Denture Technology, Faculty of Medicine, “Grigore T. Popa” University of Medicine and Pharmacy, 700115 Iași, Romania; ana.fatu@umfiasi.ro; 8 Department of Odontology, Periodontology and Fixed Prosthesis, Faculty of Medicine, “Grigore T. Popa” University of Medicine and Pharmacy, 700115 Iași, Romania; andrei.georgescu@umfiasi.ro (A.G.); mihai.vascu@umfiasi.ro (M.B.V.)

**Keywords:** negative pressure wound therapy system, open abdomen, severe acute pancreatitis, abdominal compartment syndrome

## Abstract

(1) Background: An open abdomen is a serious medical condition that requires prompt and effective treatment to prevent complications and improve patient outcomes. Negative pressure therapy (NPT) has emerged as a viable therapeutic option for temporary closure of the abdomen, offering several benefits over traditional methods. (2) Methods: We included 15 patients with pancreatitis who were hospitalized in the I–II Surgery Clinic of the Emergency County Hospital “St. Spiridon” from Iasi, Romania, between 2011–2018 and received NPT. (3) Results: Preoperatively, the mean IAP level was 28.62 mmHg, decreasing significantly postoperatively to 21.31 mmHg. The mean level of the highest IAP value recorded in pancreatitis patients treated with VAC did not differ significantly by lethality (30.31 vs. 28.50; *p* = 0.810). In vacuum-treated pancreatitis patients with a IAP level > 12, the probability of survival dropped below 50% during the first 7 days of stay in the ICU, so that after 20 days the probability of survival was approximately 20%. IAP enters the determinism of surgery with a sensitivity of 92.3% and a specificity of 99%, the cut-off value of IAP being 15 mmHg. (4) Conclusions: The timing of surgical decompression in abdominal compartment syndrome is very important. Consequently, it is vital to identify a parameter, easy to measure, within the reach of any clinician, so that the indication for surgical intervention can be made judiciously and without delay.

## 1. Introduction

AP is an acute inflammatory response of the pancreas that can be caused by various factors, including gallstones, alcohol consumption, and trauma [1]. It is characterized by a sudden onset of symptoms, such as severe abdominal pain, nausea, and vomiting, and can lead to systemic complications, such as sepsis and organ failure.

In the absence of post-necrotic damage to the gland, AP can result in complete resolution [2].

In Europe, the incidence of AP varies between 4.6 and 100 per 100,000 people and is the highest in eastern and northern Europe [3].

The 2012 revision of the Atlanta classification system identifies two phases of AP: early and late. The early phase is characterized by the development of local and systemic inflammatory responses, while the late phase is characterized by the development of complications, such as infected necrosis and organ failure. The severity of AP is classified as mild, moderate, or severe based on clinical and laboratory criteria. The mild form, also known as interstitial edematous pancreatitis, is characterized by the absence of organ failure, local or systemic complications, and usually remits within the first week of onset. Moderate AP is characterized by transitory (less than 48 h) organ failure, local complications, or exacerbation of co-morbid disease. Severe AP is characterized by persistent (more than 48 h) organ failure, which can lead to significant morbidity and mortality if not properly managed [4]. In about 80% of patients, AP is mild and self-limiting, but in up to 20% it may run a severe course with pancreatic parenchymal and/or peripancreatic tissue necrosis, responsible for substantial morbidity and a mortality rate of up to 27% [5].

Intra-abdominal hypertension (IAH) is defined as an increase in intra-abdominal pressure above 12 mmHg, which can occur in various medical conditions, including acute pancreatitis. In severe acute pancreatitis (SAP), IAH affects most patients and can lead to the development of abdominal compartment syndrome (ACS), a life-threatening condition that requires prompt intervention [6,7]. Surgical decompression for ACS in acute pancreatitis allows the release of intra-abdominal pressure and prevents further damage to organs and tissues. Following laparostomy, an open abdomen (OA) treatment is initiated, which involves the use of negative pressure wound therapy and other interventions to promote wound healing and prevent complications [8].

This technique presents several advantages by allowing the surgeon to treat or prevent IAH and to manage ACS. In addition, it dramatically decreases the operative time, reduces bleeding and provides better control of contamination, and rapid transfer of the patient to Intensive Care Unit for resuscitation. OA also allows the source of infection to be checked in the case of severe intra-abdominal sepsis (IAS) and a ‘second look’ to be planned in cases requiring a defined period of monitoring and supporting therapy [9]. In order to manage the OA, in the case of ACS, different methods are available for temporary abdominal closure: prosthetic meshes, the Bogota bag and the Wittman patch [10].

Any temporary abdominal closure device should ideally be able to contain the visceral contents, actively removing exudates, thereby quantifying the fluids lost in the third space, and also promoting granulation tissue, aiding in subsequent abdominal suturing. No temporary abdominal closure technique available to date meets all these requirements, so no gold standard technique has been imposed for the treatment of laparostomy [11].

Negative pressure therapy has established itself in the management of superficial wounds, and there is a considerable amount of data in the literature supporting the use of this therapy in temporary closure of the abdomen. However, few prospective studies evaluating the use of negative pressure therapy in achieving abdominal wall closure after surgery for intra-abdominal sepsis have been published [12].

## 2. Methods

We included 15 patients with pancreatitis who were hospitalized in the I-II Surgery Clinic of the Emergency County Hospital “St. Spiridon” from Iasi, Romania, between 2011–2018, that received NPT.

The inclusion criteria were:

- age over 18 years.
- for the Control group, from the 357 patients in the database with pancreatitis, classically treated, every 10th patient was selected, resulting in 35 patients with a primary diagnosis of pancreatitis (non-VAC group).
- for the Study group, patients treated with VAC were selected, resulting in a group of 15 people with pancreatitis (VAC group).

Patients with active bleeding and the ones who refused to participate were excluded.

The study included the demographic data of the patients, the history of the underlying diseases, and the presence of complications. The investigations performed before the start of the negative pressure therapy included laboratory samples (CBC, inflammation markers), X-rays, cultures from biological samples (wound bed or from secretions expressed before performing surgical procedures).

Monitored parameters were IAP, serum urea, serum creatinine, crystalloid and colloid intake, the PaO_2_/FiO_2_ ratio, timing of surgical intervention, and death rate.

For all patients, the NPWT device was set to a continuous aspiration type that varied between 80–140 mmHg. Pressure settings were dependent on local wound conditions, but the patient’s general status was also considered. The vacuum kit was changed at maximum 5 days.

## 3. Results

The age of the patients varied from 24 to 89 years, the average of the group being 56.80 years ± 17.83, with a homogeneous distribution.

**Figure 1:**
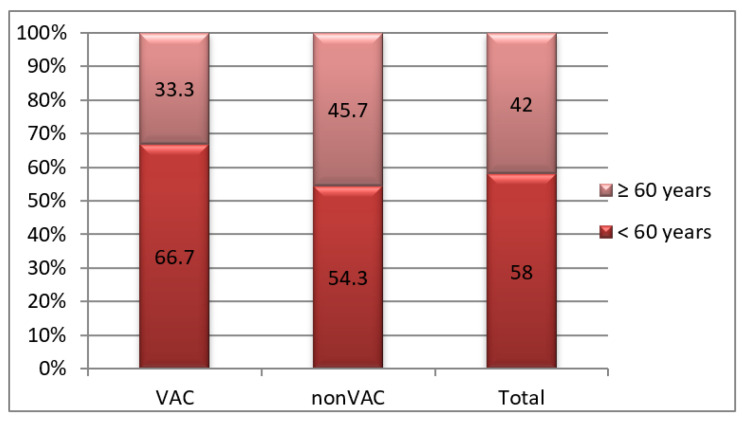
Distribution by age and treatment methods.

The distribution of cases by age groups and treatment methods was homogeneous, although the proportion of pancreatitis cases treated with VAC was moderately higher at ages under 60 years. The same aspect is noted in patients with classic treatment (66.7% vs. 54.3%; *p* = 0.311) (Figure 1)

The gender distribution highlighted higher frequencies in men (64% of the total study group), sex ratio M/F = 1.8/1 and was homogeneous depending on the treatment method (66.7% vs. 62.9%; *p* = 0.311).

**Figure 2:**
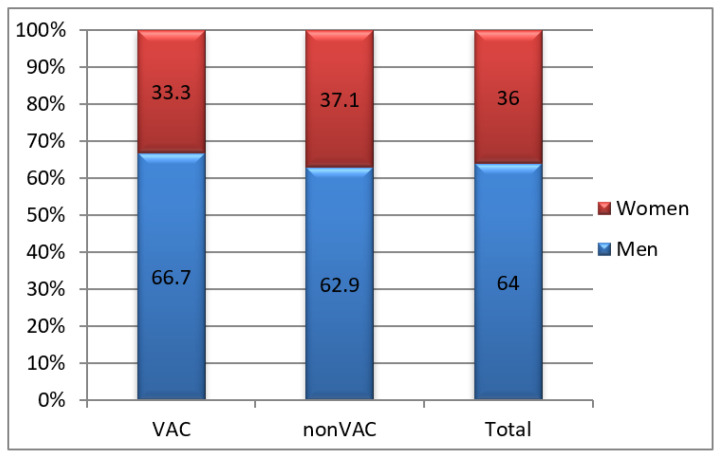
Group distribution by gender.

In classically treated patients, the mean age was significantly lower in men (50.0 vs. 67.85 years; *p* = 0.007), while in patients treated with VAC, the difference was not statistically significant (55.90 vs. 59.80 years; *p* = 0.622) (Figure 2).

### 3.1. IAP (Intra-Abdominal Pressure)

**Figure 3:**
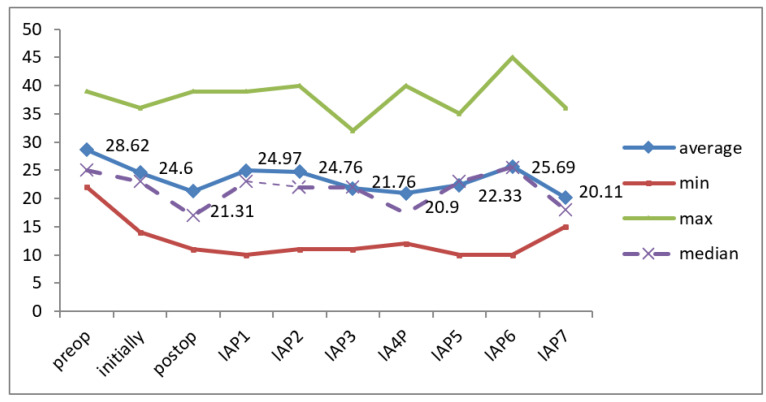
IAP variation during monitoring.

The recorded values for intra-abdominal pressure (IAP) showed a median value that was close to the mean level, and the results of the Skewness test indicated that the values were evenly distributed within the range of [−2 ÷ 2]. This suggests that the recorded values were homogeneous, so significance tests for continuous variables can be used. Preoperatively, the mean IAP level was 28.62 mmHg, decreasing significantly postoperatively to 21.31 mmHg (*p* = 0.002). At the last assessment, the IAP level was significantly lower in patients remaining in the study compared to baseline (24.60 vs. 20.11 mmHg; *p* = 0.002) (Table 1, Figure 3).

**Figure 4:**
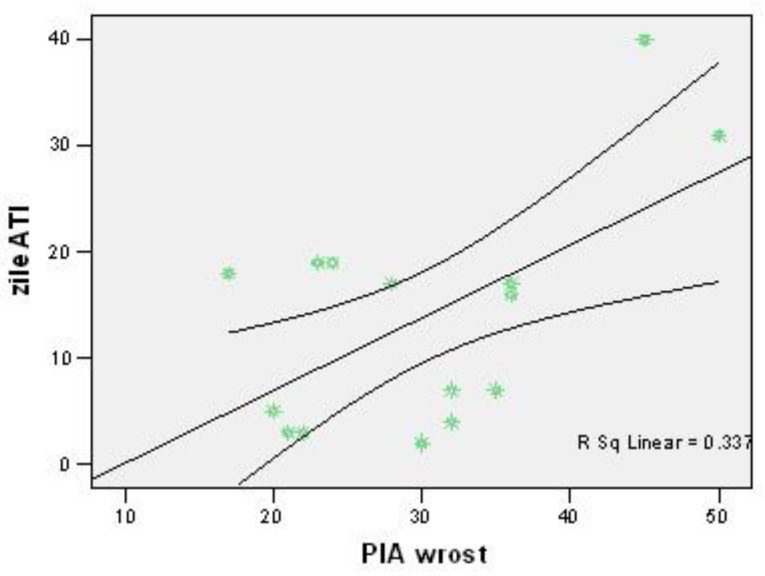
The correlation between the highest IAP values with the number of days spent in ICU. Each graphic symbol represents one of the 15 case studies included in the group of admitted patients.

In our study, 58% of patients showed a correlation between higher intra-abdominal pressure (IAP) values and longer periods of hospitalization in the intensive care unit (ICU) (r = +0.581; *p* = 0.023) (Figure 4).

The mean level of the highest IAP value recorded in pancreatitis patients treated with VAC did not differ significantly by lethality (30.31 vs. 28.50; *p* = 0.810).

### 3.2. Water Balance (WH)

Our results highlighted the homogeneity of the values recorded for the water balance (Table 2, Figure 3).

**Figure 5:**
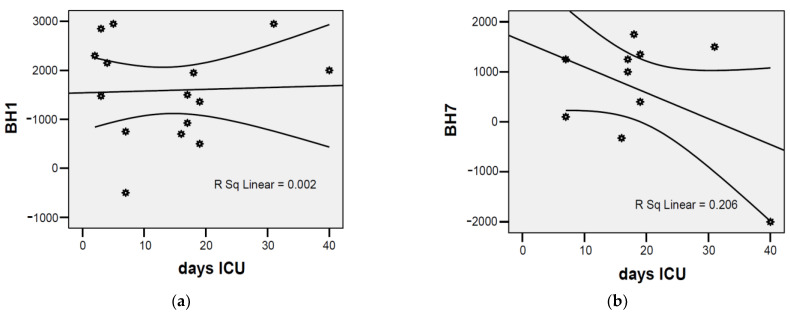
The correlation between the water balance and the number of days of stationary in ICU: (**a**) first evaluation; (**b**) seventh evaluation. Each graphic symbol represents one of the 15 case studies included in the group of admitted patients.

The water balance at the first evaluation and the number of days of hospitalization in the ICU were apparently independent parameters (r = +0.040; *p* = 0.889); however, at the seventh evaluation, approximately 45% of the patients recorded lower values of the balance hydric if the stay in ICU was longer (r = −0.454; *p* = 0.048) (Figure 5).

### 3.3. Serum Urea

In pancreatitis patients treated with VAC, peak urea levels ranged from 17–228 mg/dL, with a mean of 113.40 mg/dL ± 64.51 close to the median value (97 mg/dL).

**Figure 6:**
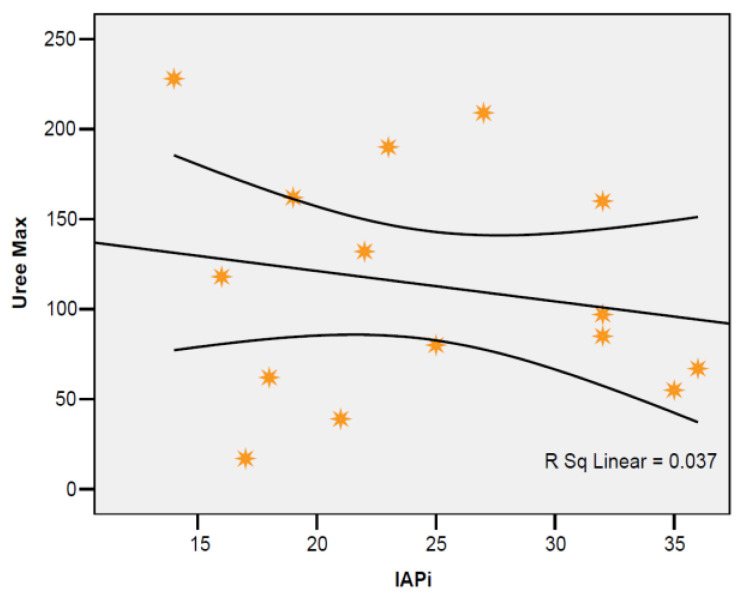
Correlation of IAP at first assessment with peak urea level. Each graphic symbol represents one of the 15 case studies included in the group of admitted patients.

The correlation between IAP at the first assessment and peak serum urea level was indirect, low in intensity, but the result cannot be extrapolated to the general population (r = −0,191; *p* = 0,495) (Figure 6).

### 3.4. Serum Creatinine

**Figure 7:**
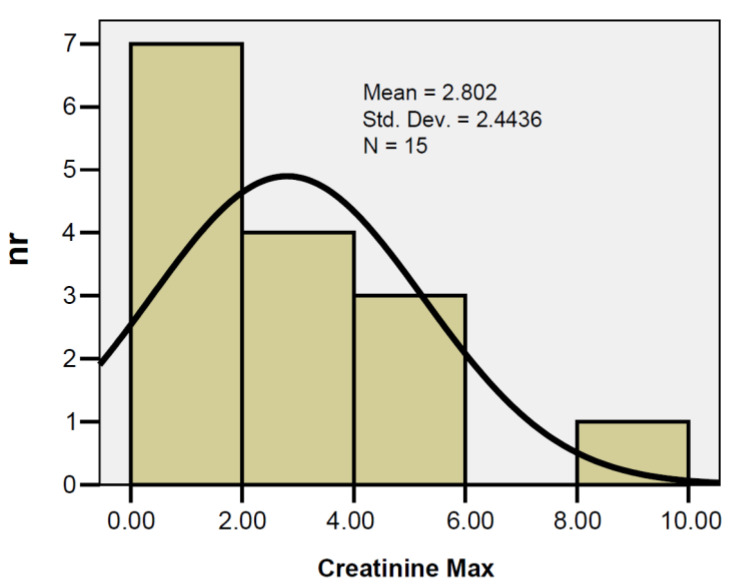
Distributions of serum creatinine values.

In pancreatitis patients treated with VAC, peak creatinine ranged from 0.59 to 9.90 mg/dL, with a mean of 2.80 mg/dL ± 2.44 away from the median value (2.07 mg/dL), and the Skewness test result = 2.011 suggests that the variable was non-continuous (Figure 7).

**Figure 8:**
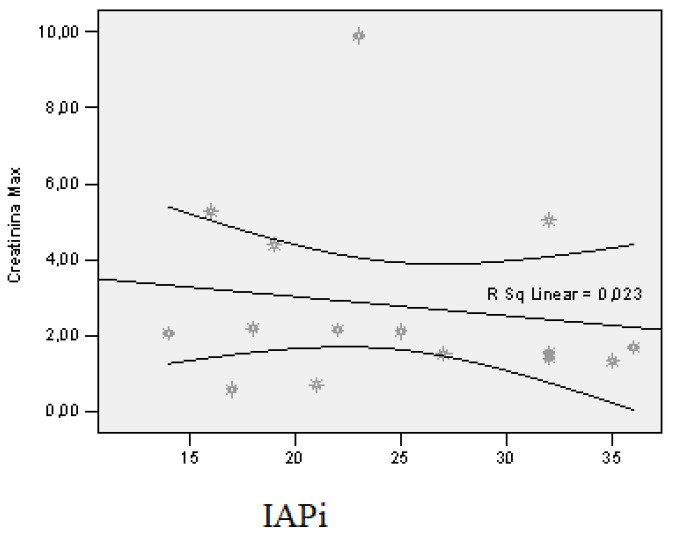
Correlation of PIA at first assessment with peak creatinine level. Each graphic symbol represents one of the 15 case studies included in the group of admitted patients.

The correlation between IAP at first assessment and peak serum creatinine was indirect, low in intensity, but the result cannot be extrapolated to the general population (r = −0.151; *p* = 0.592) (Figure 8).

### 3.5. Crystalloid

**Figure 9:**
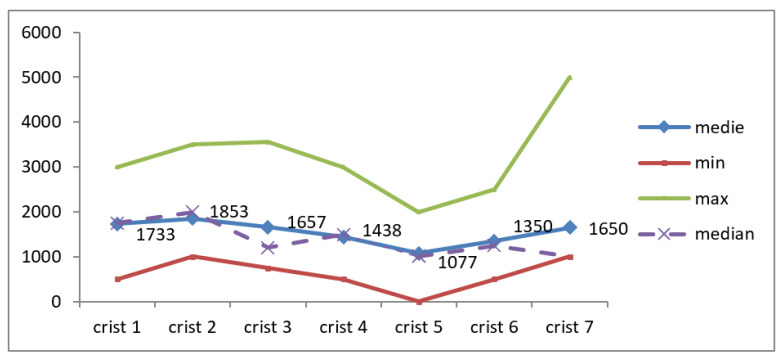
Variation of crystalloids during monitoring.

The data obtained regarding crystalloid administration are illustrated in Table 3 and Figure 9.

The correlations between crystalloids at the first assessment with the duration of hospitalization (r = −0.112; *p* = 0.692), as well as with the number of days of hospitalization in the ICU (r = −0.211; *p* = 0.451), were indirect, low in intensity, non-significant from a statistical point of view.

The correlation between IAP and crystalloid at first assessment was indirect, low in intensity, but the result cannot be extrapolated to the general population (r = −0.397; *p* = 0.143).

### 3.6. Colloids

**Figure 10:**
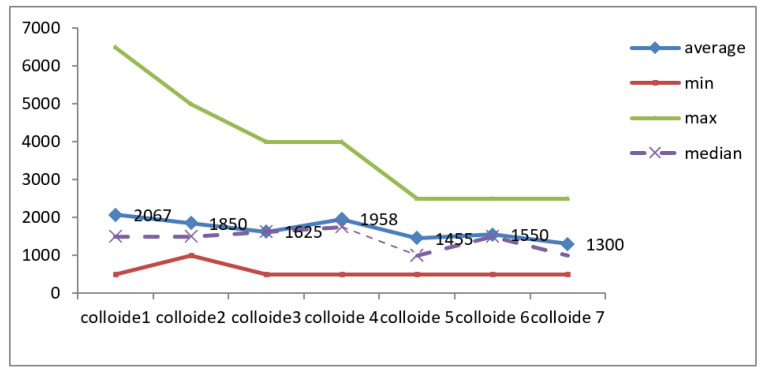
Colloid variation during monitoring.

In the case of colloids as well, we observe the homogeneity of the values (Figure 10).

### 3.7. PaO_2_/FiO_2_

**Figure 11:**
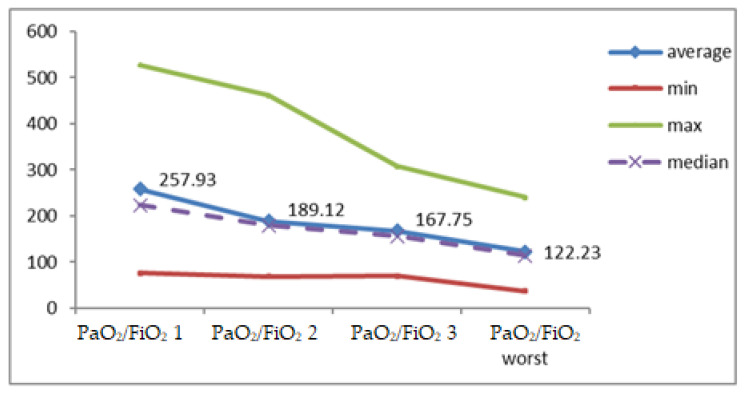
Variation of PaO_2_/FiO_2_ ratio during monitoring.

At the first assessment, the PaO_2_/FiO_2_ ratio ranged from 76 to 528, with a mean level of 257.93 ± 135.76, and significant decreases (1–3) during follow-up to a mean level of 167.75 ± 76.77 (*p* = 0.001) (Table 4, Figure 11).

The correlations between the lowest PaO_2_/FiO_2_ ratio with the duration of hospitalization (r = +0.061; *p* = 0.829), as well as with the number of days of hospitalization in the ICU (r = −0.185; *p* = 0.509) were not significant from the statistical view.

The correlation of IAP at the first assessment and worst PaO_2_/FiO_2_ ratio was direct, low in intensity, but statistically insignificant (r = +0.130; *p* = 0.644).

### 3.8. Evaluation of the Indication for Surgical Intervention

In patients with pancreatitis treated with vacuum therapy, surgical interventions were performed in 86.7% of cases. Analysis of the ROC curve revealed that the initial level of intra-abdominal pressure (IAP) was a significant determinant of the need for surgery (area under the curve [AUC] = 0.962; 95% confidence interval [CI]: 0.858–1.065), with a sensitivity of 92.3% and a specificity of 99%. The optimal cut-off value for IAP was 15 mmHg. These findings suggest that monitoring of IAP levels in pancreatitis patients treated with vacuum therapy may help identify those who are at risk of requiring surgical intervention.

**Figure 12:**
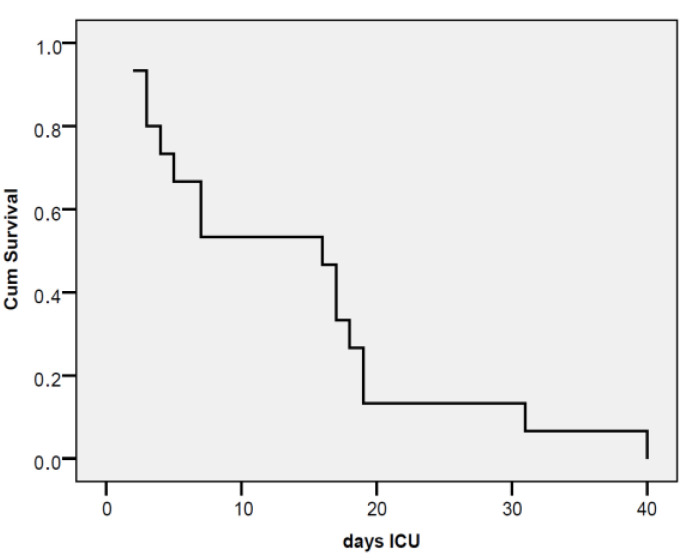
Kaplan-Meier survival curve in vacuum-treated pancreatitis patients with a PIA level > 12.

In patients with pancreatitis treated with vacuum therapy and an intra-abdominal pressure (IAP) level greater than 12 mmHg, the probability of survival drops below 50% within the first 7 days of ICU admission. After 20 days, the probability of survival is approximately 20%. (Figure 12).

**Figure 13:**
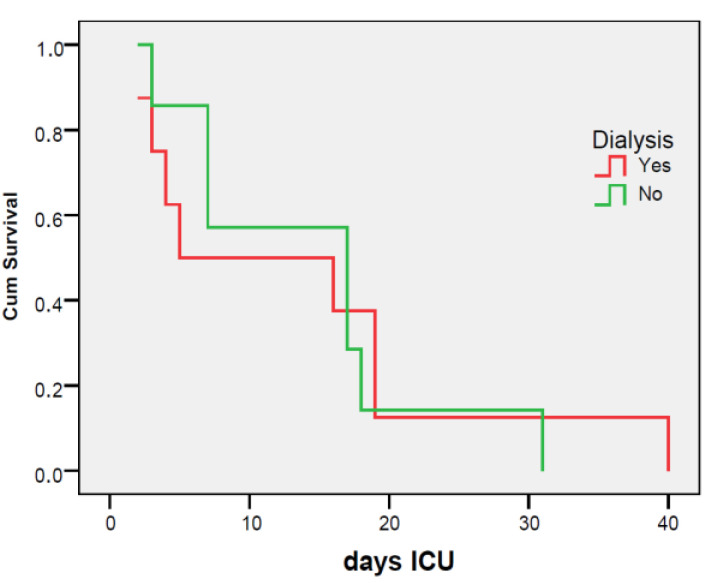
Kaplan-Meier survival curve in patients with pancreatitis on dialysis, treated with vacuum with a level of PIA > 12.

Patients with pancreatitis who are treated with vacuum therapy and have an intra-abdominal pressure (IAP) level greater than 12 mmHg and require dialysis have a poor prognosis. The probability of survival drops below 50% within the first 5 days of ICU admission (Figure 13).

### 3.9. Death Rate

**Figure 14:**
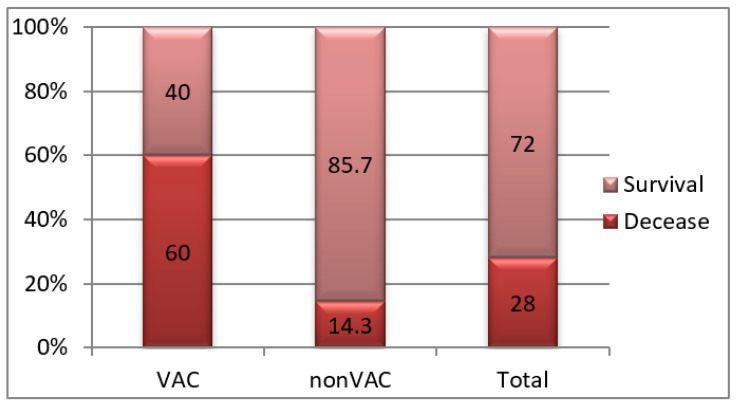
Batch structure according to death.

Overall, in the study group, death occurred in 28% of patients with pancreatitis, in 60% of pancreatitis patients treated with VAC and in only 14.3% of those treated classically (*p* = 0.002) (Figure 14).

Among the monitored parameters, urea and creatinine are good predictors of death (AUC > 0.600) (Table 5).

## 4. Discussion

### 4.1. IAP

Preoperatively, the mean IAP level was 28.62 mmHg, decreasing significantly postoperatively to 21.31 mmHg (*p* = 0.002). At the last evaluation, the IAP level was significantly lower in patients remaining in the study compared to the initial moment (24.60 vs. 20.11 mmHg; *p* = 0.002).

Approximately 58% of patients associated higher IAP values with more days of ICU stay (r = +0.581; *p* = 0.023). The mean level of the highest IAP value recorded in patients with pancreatitis treated with VAC did not differ significantly by mortality (30.31 vs. 28.50; *p* = 0.810).

As we expected, the mean preoperative level of IAP was 28.62, decreasing significantly postoperatively, so that, at the last evaluation, the values were greatly diminished (*p* = 0.002) and the number of days of stay in the intensive care unit was statistically significantly associated with increased IAP values. The World Society of the Abdominal Compartment Syndrome (WSACS) makes a distinction between IAH and ACS. IAH is defined as IAP > 12 mmHg without associated organ failure. ACS is defined by IAP ≥ 20 mmHg in association with failure of at least one organ system [8].

The timing of surgical decompression is a topic of interest these days. Concurrent respiratory failure is thought to be due to the transmission of IAH to the thoracic compartment. IAH causes diaphragmatic elevation, compression and decreased lung compliance of the lungs, and atelectasis.

Inferior vena cava compression caused by IAH decreases venous return. The combination of these phenomena generates increased oxygen requirements as well as a “shunt effect”, exposing the patient to a risk of hypoxemia and hypocapnia. From a hemodynamic point of view, IAH increases systemic and pulmonary vascular resistance. These combined phenomena explain the risk of peripheral hypoperfusion, acute renal failure and intestinal ischemia. In addition, decreased venous return in association with increased cardiac afterload increases the risk of heart failure [13,14].

It should not be surprising that, in cases of prolonged exposure to a high IAP, organ function is irreversibly damaged, but the exact point in time when decompressive laparotomy can have maximal results is difficult to determine.

The combination of decompressive laparotomy and negative pressure therapy has been shown to effectively reduce intra-abdominal pressure, improve visceral perfusion, and decrease the transmission of inflammatory mediators into the bloodstream [15]. These interventions can lower the risk of developing multiorgan dysfunction due to sepsis, which is a serious complication of acute pancreatitis.

Although surgical decompression and an open abdomen can save life, the gesture can lead to some complications, such as: stimulation of a hypercatabolic state and loss of proteins through the elimination of peritoneal fluid, development of fistula, large ventral hernia or hemorrhagic complications, which can be life-threatening dangers, including reperfusion syndrome [16].

In patients treated with negative pressure, mortality was not significantly influenced by the mean IAP value. 

This may be due to another secondary consequence, such as SAP, which isn’t related to an elevated IAP, or to the patient’s presence of a comorbidity that has been out of balance as a result of the progression of pancreatitis.

By plotting the ROC curve, it is demonstrated that the initial level of IAP enters in the determinism of surgery (AUC = 0.962; CI95%: 0.858–1.065) with a sensitivity of 92.3% and a specificity of 99%, the cut-off value of IAP being 15 mmHg.

There is evidence that early decompression may improve survival because IAH adds to the impaired organ function in patients with SAP. However, other reasons for organ dysfunction are present in patients with IAH, and it is logical that decompression could not completely reverse this process. [17]

It is important that we can identify a parameter, easy to measure, within the reach of any clinician, so that the indication for surgical intervention can be made judiciously and without delay.

In vacuum-treated pancreatitis patients with an IAP level > 12, the probability of survival drops below 50% during the first 7 days of ICU stay, so that after 20 days the probability of survival is approximately 20%.

It is emphasized that an increased value of intra-abdominal pressure, maintained for a long period of time, without being able to be remedied by various specific treatment methods, has a statistically significant influence, in terms of short and long-term survival, observing that the rate drops significantly, from 50% in the first week to only 20% in the third week [18].

### 4.2. The Water Balance

The median value close to the mean value and the results of the Skewness test in the interval [−2 ÷ 2] highlighted the homogeneity of the series of values recorded for the water balance. The water balance at the first evaluation and the number of days of hospitalization in the ICU were apparently independent parameters (r = +0.040; *p* = 0.889); however, at the seventh evaluation, approximately 45% of the patients recorded lower values of the balance hydric, if the stay in ICU was longer (r = −0.454; *p* = 0.048).

The water balance at the first evaluation and the number of days of hospitalization in the ICU were apparently independent parameters (r = +0.040; *p* = 0.889); however, at the seventh evaluation, approximately 45% of the patients recorded lower values of the balance hydric, if the stay in ICU was longer (r = −0.454; *p* = 0.048).

Consistent with existing literature, the water balance level exhibited a downward trend during the treatment course, which is considered the optimal approach for fluid resuscitation in cases of intra-abdominal hypertension/abdominal compartment syndrome. This is because maintaining a proper fluid balance is a critical factor in reducing the risk of these conditions. In a prospective cohort of AP patients, those who re-received more than 4.1 L of fluids in 24 h presented more organ failure, fluid collections, and respiratory and renal insufficiency [19].

We obtain similar results regarding the improvement of the biological parameters. According to the recommendations in the literature, its lower values are recorded if the patient stayed longer in the intensive care unit, where the care, respectively the evaluation of this parameter, is much stricter [20,21].

Due to numerous mechanisms, hypovolemia is a well-recognized risk factor of poor outcome in patients with AP [22]. During severe AP, an uncontrolled inflammatory response alters endothelial functions, leading to vasodilation, capillary leakage and edema. Together with vomiting, ascite or ileus, this vascular dysfunction promotes hypovolemia and acute circulatory failure.

Circulatory dysfunction leads to tissue hypoperfusion, ischemia and subsequently to self-sustaining disease with persistent pancreatic injury, extra-pancreatic tissue damage and organ failures [23]. Supportive care, with the use of intravenous fluid hydration, is a mainstay of acute pancreatitis treatment in the first 12–24 h.

The median value close to the mean value and the results of the Skewness test in the interval [−2 ÷ 2], except for the last determination, highlighted the homogeneity of the series of values recorded for crystalloids.

The correlations between crystalloids at the first assessment with the duration of hospitalization (r = −0.112; *p* = 0.692), as well as with the number of days of hospitalization in the ICU (r = −0.211; *p* = 0.451) were indirect, reduced in intensity, and insignificant from a statistical point of view. The correlation between IAP and crystalloids at the first assessment was indirect, low in intensity, but the result cannot be extrapolated to the general population (r = −0.397; *p* = 0.143).

Except for the first two determinations, the median value close to the mean value and the results of the Skewness test in the interval [−2 ÷ 2] highlighted the homogeneity of the series of values recorded for colloids.

The correlations between colloids at the first evaluation with the duration of hospitalization (r = −0.130; *p* = 0.645), as well as with the number of days of hospitalization in the ICU (r = −0.051; *p* = 0.857), were indirect, low in intensity, and non-significant from a statistical point of view. IAP and colloids at first assessment were apparently independent parameters (r = −0.001; *p* = 0.998).

Determining the ideal fluid for resuscitation in acute pancreatitis remains an ongoing challenge. The initial studies on fluid resuscitation have shown varied results, which could be attributed to the use of different types of fluids (colloids and crystalloids). Further research is needed to determine which fluid type is most effective for resuscitation in acute pancreatitis.

Colloids have been shown to be superior to crystalloids in animal experiments [24,25], which may be since they are not as permeable to leakage in pancreatic microcirculation as crystalloids.

### 4.3. Acute Renal Failure

One of the common complications of severe acute pancreatitis in critically ill patients with intra-abdominal hypertension, septic shock and/or abdominal compartment syndrome is acute kidney injury [21]. The presence of acute kidney injury means a higher risk of morbidity and mortality and also leads to an increase in the economic cost of treatment [26].

In our study, in pancreatitis patients treated with negative pressure therapy, the maximum urea level recorded values in the range of 17–228 mg/dL, with a mean of 113.40 mg/dL ± 64.51, close to the median value (97 mg/dL). The correlation between IAP at the first assessment with the maximum level of serum urea was indirect, and low in intensity; the result could not be extrapolated to the general population (r = −0.191; *p* = 0.495).

In pancreatitis patients treated with VAC, peak creatinine ranged from 0.59 to 9.90 mg/dL, with a mean of 2.80 mg/dL ± 2.44 away from the median value (2.07 mg/dL). The correlation of PIA at the first assessment with peak serum urea was indirect, but the result cannot be extrapolated to the general population (r = −0.151; *p* = 0.592).

The prevalence of acute renal failure in patients admitted to intensive care units with acute pancreatitis (15.05%) even exceeded the prevalence of renal failure in sepsis (13.2%) [27].

In our study, we obtain data similar to those reported in the literature [28,29,30], regarding the increase in mortality in patients associating renal insufficiency, the average level of creatinine correlating with mortality (*p* = 0.045).

In the present study, there is a lower number of days of hospitalization in patients with a higher creatinine level (r = −0.169; *p* = 0.532), and in those with respiratory failure a greater number of days of stay in the ICU (r = −0.173; *p* = 0.523).

### 4.4. Respiratory Failure

#### PaO_2_/FiO_2_

The median value close to the mean level and the results of the Skewness test in the range [−2 ÷ 2] highlighted the homogeneity of the series of recorded values for the PaO_2_/FiO_2_ ratio, so significance tests for continuous variables can be used.

At the first assessment, the PaO_2_/FiO_2_ ratio ranged from 76–528, recording a mean level of 257.93 ± 135.76, with significant decreases during monitoring to a mean level of 167.75 ± 76.77 (*p* = 0.001). The correlations between the lowest PaO_2_/FiO_2_ ratio with the duration of hospitalization (r = +0.061; *p* = 0.829), as well as with the number of days of hospitalization in the ICU (r = −0.185; *p* = 0.509) were not significant from the statistical view.

The correlation of IAP at the first assessment and the lowest PaO_2_/FiO_2_ ratio was direct, low in intensity, but not statistically significant.

Limitations and potential errors may result from the behavior and non-compliance of the patients with the medical advice, the limited sample of patients, the interpretation of CT, MRI imaging by several radiologists, as well as the timing of the surgery.

## 5. Conclusions

The timing of surgical decompression in abdominal compartment syndrome is a topic of interest these days.

It is important that we can identify a parameter, easy to measure, within the reach of any clinician, so that the indication for surgical intervention can be made judiciously and without delay.

There is evidence that early decompression may improve survival, because IAH adds to the impaired organ function in patients with SAP. However, other reasons for organ dysfunction are present in patients with IAH, and it is logical that decompression could not completely reverse this process.

## Figures and Tables

**Table 1:** Descriptive data IAP (mmHg) (evaluation 1–7).

Statistical Indicators		Preop	Initially	Postop	IAP1	IAP2	IAP3	IAP4	IAP5	IAP6	IAP7
Number		13	15	13	15	15	13	12	9	10	9
Average		28.62	24.60	21.31	24.97	24.76	21.76	20.90	22.33	25.69	20.11
Median		25.00	23.00	17.00	23.00	22.00	22.00	17.43	23.00	25.50	18.00
Standard Dev.		5.80	7.32	8.86	9.64	8.31	5.59	9.57	7.43	10.14	6.66
Alternative		20.27	29.76	41.58	38.61	33.56	25.69	45.79	33.27	39.47	33.12
Skewness Test		0.761	0.218	0.839	0.109	0.390	−0.145	1.352	0.068	0.427	1.912
Er.std Skewness		0.616	0.580	0.616	0.580	0.580	0.616	0.637	0.717	0.687	0.717
Minimum		22	14	11	10.0	11.0	11.0	12.0	10	10	15
Maximum		39	36	39	39.0	40.0	32.0	40.0	35	45	36
Percentile	25	25	18	15	16	20	18	14	17	17	16
	50	25	23	17	23	22	22	17	23	26	18
	75	34	32	28	35	30	26	25	28	32	23

**Table 2:** Descriptive water balance data (evaluation 1–7).

Statistical Indicators		BH1	BH2	BH3	BH4	BH5	BH6	BH7
Number		15	15	14	12	10	10	10
Average		1591	1071	992	1596	925	1213	628
Median		1500	1500	858	2013	725	1263	1125
Standard Dev.		999	1409	821	1228	1251	938	1135
Alternative		62.79	131.56	82.76	76.94	135.24	77.33	180.73
Skewness Test		−0.362	−1.179	0.243	−0.753	0.123	−0.287	−1.535
Er.std Skewness		0.580	0.580	0.597	0.637	0.687	0.687	0.687
Minimum		−500	−2600	−500	−1000	−850	−375	−2000
Maximum		2950	3200	2500	3130	2800	2250	1750
Percentile	25	750	200	438	550	38	475	−6
	50	1500	1500	858	2013	725	1263	1125
	75	2300	1975	1631	2538	2238	2231	1388

**Table 3:** Crystalloid descriptive data (evaluation 1–7).

Statistical Indicators		Cryst 1	Cryst 2	Cryst 3	Cryst 4	Cryst 5	Cryst 6	Cryst 7
Number		15	15	14	12	11	10	10
Average		1733	1853	1657	1438	1077	1350	1650
Median		1750	2000	1200	1500	1000	1250	1000
Standard Dev.		759	778	870	692	575	580	1270
Alternative		43.80	41.99	52.50	48.12	53.39	42.96	76.97
Skewness Test		0.232	0.826	1.030	0.836	0.094	0.727	2.476
Er.std Skewness		0.580	0.580	0.597	0.637	0.661	0.687	0.687
Minimum		500	1000	750	500	0	500	1000
Maximum		3000	3500	3550	3000	2000	2500	5000
Percentile	25	1000	1000	1000	1000	1000	1000	1000
	50	1750	2000	1200	1500	1000	1250	1000
	75	2250	2000	2125	1500	1350	1625	1750

**Table 4:** Descriptive data PaO2/FiO2.

Statistical Indicators		PaO_2_/FiO_2_ 1	PaO_2_/FiO_2_ 2	PaO_2_/FiO_2_ 3	PaO_2_/FiO_2_ Worst
Number		15	15	15	15
Average		257.93	189.12	167.75	122.23
Median		223.00	179.50	156.00	114.00
Standard Dev.		135.76	99.76	76.77	62.43
Alternative		52.63	52.75	45.76	51.08
Skewness Test		0.660	1.507	0.849	0.323
Er.std Skewness		0.580	0.580	0.580	0.580
Minimum		76	68.80	70.00	37.20
Maximum		528	461	308	240
Percentile	25	160	110	111	69
	50	223	180	156	114
	75	327	220	187	175

**Table 5:** Laboratory parameters, predictors of death.

Test Result Variable	Area	Std. Error ^a^	Asymptotic Sig ^b^	Asymptotic 95% CI	
				Lower Bound	Upper Bound
PIAi	0.423	0.305	0.734	−0.175	1.022
PIA worst	0.519	0.249	0.932	0.030	1.008
BH1	0.538	0.138	0.267	0.267	0.809
Uree Max	0.885	0.109	0.672	0.672	1.097
Creatinine Max	0.808	0.159	0.496	0.496	1.120
PaO_2_/FiO_2_ worst	0.385	0.139	0.111	0.111	0.658

^a^ Under the nonparametric assumption, ^b^ Null hypothesis: true area = 0.5.

## Data Availability

The data published in this research are available on request from the first author and corresponding authors.
